# Reprogramming of adult human dermal fibroblasts to induced dorsal forebrain precursor cells maintains aging signatures

**DOI:** 10.3389/fncel.2023.1003188

**Published:** 2023-01-30

**Authors:** Amy McCaughey-Chapman, Marta Tarczyluk-Wells, Catharina Combrinck, Nicole Edwards, Kathryn Jones, Bronwen Connor

**Affiliations:** ^1^Department of Pharmacology and Clinical Pharmacology, Centre for Brain Research, School of Medical Science, Faculty of Medical and Health Sciences, University of Auckland, Auckland, New Zealand; ^2^School of Biological Sciences, Faculty of Science, University of Auckland, Auckland, New Zealand

**Keywords:** human induced dorsal forebrain precursors, direct cell reprogramming, induced pluripotent stem cell, neurodegenerative disease, senescence, telomere length, oxidative stress, DNA methylation

## Abstract

**Introduction:** With the increase in aging populations around the world, the development of *in vitro* human cell models to study neurodegenerative disease is crucial. A major limitation in using induced pluripotent stem cell (hiPSC) technology to model diseases of aging is that reprogramming fibroblasts to a pluripotent stem cell state erases age-associated features. The resulting cells show behaviors of an embryonic stage exhibiting longer telomeres, reduced oxidative stress, and mitochondrial rejuvenation, as well as epigenetic modifications, loss of abnormal nuclear morphologies, and age-associated features.

**Methods:** We have developed a protocol utilizing stable, non-immunogenic chemically modified mRNA (cmRNA) to convert adult human dermal fibroblasts (HDFs) to human induced dorsal forebrain precursor (hiDFP) cells, which can subsequently be differentiated into cortical neurons. Analyzing an array of aging biomarkers, we demonstrate for the first time the effect of direct-to-hiDFP reprogramming on cellular age.

**Results:** We confirm direct-to-hiDFP reprogramming does not affect telomere length or the expression of key aging markers. However, while direct-to-hiDFP reprogramming does not affect senescence-associated β-galactosidase activity, it enhances the level of mitochondrial reactive oxygen species and the amount of DNA methylation compared to HDFs. Interestingly, following neuronal differentiation of hiDFPs we observed an increase in cell soma size as well as neurite number, length, and branching with increasing donor age suggesting that neuronal morphology is altered with age.

**Discussion:** We propose direct-to-hiDFP reprogramming provides a strategy for modeling age-associated neurodegenerative diseases allowing the persistence of age-associated signatures not seen in hiPSC-derived cultures, thereby facilitating our understanding of neurodegenerative disease and identification of therapeutic targets.

## 1. Introduction

The process of aging affects all tissues of the body making aging a primary risk factor for many human pathologies and the development of numerous neurodegenerative diseases. While neurodegenerative disorders such as Alzheimer’s disease or Parkinson’s disease can emerge midlife (early-onset familial versions), the majority of cases are sporadic and develop in older age. Since the majority of neurons are born during embryogenesis and there is a limited capacity for new neurons to be born later in life, these cells are particularly affected by cellular aging. Aging also leads to a decline in neuronal plasticity and cognitive performance in the majority of healthy people.

Our progress and understanding of the molecular basis of neurodegenerative disorders to date has been impeded by the limited accessibility of human brain tissue and difficulties in obtaining live brain cells from patients. To date, the majority of studies have been restricted to the use of animal models that, while generating useful data, have limitations in regard to the study and treatment of human disease. However, since the discovery that adult human dermal fibroblasts (HDFs) can be reprogrammed to generate induced pluripotent stem cells (hiPSCs; Takahashi et al., 2007), and that these can be induced towards a neural stem cell fate with subsequent generation of mature neurons and glia, there has been a rapid increase in the use of hiPSC technology to model neurodegenerative disease. There has been considerable success in modeling early developmental disorders (Ebert et al., 2009; Lee et al., 2009) and early-onset neurodegenerative diseases such as familial Alzheimer’s disease (Israel et al., 2012; Shi et al., 2012). However, modeling of late-onset neurodegenerative diseases has often failed to recapitulate significant features of the disease phenotype (Srikanth and Young-Pearse, 2014; Wu et al., 2019).

One of the major limitations in using hiPSC technology to model diseases of aging is that reprogramming fibroblasts to a pluripotent stem cell state erases age associated features. This results in the production of cells that show the behavior of an embryonic stage exhibiting longer telomeres, reduced oxidative stress, and/or mitochondrial rejuvenation (Suhr et al., 2009, 2010; Lapasset et al., 2011). Moreover, hiPSCs derived from fibroblasts obtained from older patients no longer present abnormal nuclear morphologies and age-associated features such as DNA damage increased DNA methylation, increased reactive oxygen species, reduced levels of nuclear organization proteins or loss of heterochromatin markers (Horvath, 2013; Miller et al., 2013; Lo Sardo et al., 2017; Strässler et al., 2018). This confirmed prior research which suggested that biological age and developmental state are separable (Chen and Skutella, 2022). In contrast, the generation of human neurons by direct reprogramming of fibroblasts retains age-specific transcriptional profiles, keeping the reprogrammed cells at the same “biological age” as the donor cells (Mertens et al., 2015; Yang et al., 2015).

We have developed a novel protocol that utilizes stable and non-immunogenic, chemically modified mRNA (cmRNA) to convert adult HDF to induced dorsal forebrain precursor cells (hiDFPs; Edwards et al., 2022). By transiently over-expressing the neural genes *SOX2* and *PAX6* using cmRNA we can generate hiDFPs which can subsequently be differentiated into cortical neurons (Maucksch et al., 2012; Connor et al., 2018; Playne et al., 2018; Edwards et al., 2022). Following 21 days of reprogramming, we have demonstrated that hiDFPs express the dorsal forebrain markers* FOXG1, NGN2, BRN2, TBR1, TBR2, DLX2, CTIP2*, and *SATB2* (Edwards et al., 2022). Following differentiation, hiDFP-derived neurons expressed *TBR1, CUX1, TUJ1, MAP2* and *VGLUT1*, with a down-regulation of *CTIP2*. Immunocytochemical analysis demonstrated ~85% of TUJ1+ neurons co-expressed vGLUT1 following hiDFP differentiation. Furthermore, functionality was demonstrated by live-cell calcium imaging (Edwards et al., 2022). As this method does not pass through a pluripotency state, we hypothesize that our direct-to-hiDFP reprogramming method will prevent the cell rejuvenation observed in hiPSCs and maintain the aging phenotype of the starting HDFs, providing a better mechanism by which to model neurological diseases of aging.

By analysis of an array of aging biomarkers, this study demonstrates for the first time the effect of direct-to-hiDFP reprogramming on cellular age. Our findings show that cmRNA direct-to-hiDFP reprogramming does not affect telomere length or the expression of key aging markers. However, while cmRNA direct-to-hiDFP reprogramming does not affect senescence-associated β-galactosidase (SA-βgal) activity, it does enhance the level of mitochondrial reactive oxygen species (ROS) and DNA methylation as determined by the production of 5-methylcytosine (5-mC). We also assessed the maturation efficiency of hiDFPs to neurons and observed an increase in cell soma size as well as neurite number, length, and branching with donor age.

## 2. Materials and methods

### 2.1. Cell lines

Nine adult human dermal fibroblast cell lines (HDFs) were obtained from Cell Applications Inc or The NINDS Human Cell and Data Repository. Cell lines were split into three groups based on the age of the donor: 20/30s (26, 33, and 35 years old), 50s (50, 55, and 55 years old) and 70s (70, 73, and 76 years old). RNA and DNA from HDFs and their corresponding induced pluripotent stem cell lines (hiPSCs; *n* = 3, average age 57 years) were kindly donated by Prof Alice Pebay from the University of Melbourne and Prof Alan Davidson from the University of Auckland.

### 2.2. Cell culture, reprogramming, and cortical differentiation

HDFs were cultured according to the protocol from the supplier. Cells were grown either in DMEM (Thermo Fisher Scientific) supplemented with 10% fetal bovine serum (FBS), or alpha MEM (Thermo Fisher Scientific) supplemented with 10% FBS. *SOX2* and *PAX6* cmRNA were designed and manufactured by Ethris GmbH (Connor et al., 2018).

Reprogramming was undertaken as previously described in Edwards et al. (2022). Briefly, prior to *SOX2* and *PAX6* cmRNA transfection, cells were plated at a density of 300,000 cells per well on a 6-well plate. On the first day of transfection the media was changed to GYN cortical reprogramming medium consisting of Neurobasal-A (NBA; Thermo Fisher Scientific) with 1 mM valproic acid, 1% penicillin/streptomycin/glutamine, 2% B-27 with vitamin-A (Thermo Fisher Scientific), 20 ng/ml epidermal growth factor (EGF; Prospec Bio), 20 ng/ml fibroblast growth factor 2 (FGF2; Prospec Bio), 2 μg/ml heparin (Sigma), 5 μM Gö6983 (Abcam), 10 μM Y27632 (Abcam) and 1% N-2 supplement (Thermo Fisher Scientific). Chemically modified mRNA (2.5 μg cmRNA) transfections were performed using Lipofectamine RNAiMAX (Life Technologies) in OptiMem media (Thermo Fisher Scientific). Transfections were performed in four consecutive cycles with 5-h incubations. Three days after the final transfection, cells were passaged and fed with GYN cortical reprogramming medium every other day. From Day 7 the GYN cortical reprogramming medium also included 1 μM RepSox. Subsequent passages were performed every 7 days until full hiDFP cell fate was achieved at Day 21.

Neuronal differentiation was undertaken as previously described in Edwards et al. (2022). Briefly, neurons were derived from reprogrammed hiDFPs by seeding 80,000 cells/well in a 24-well plate onto GelTrex-coated glass coverslips. Cells were cultured for 14 or 19 days in NBA-based cortical differentiation medium containing 1% penicillin-streptomycin, 2% B-27 supplement with retinoic acid, 1% N-2 supplement, 10 mM Y-27632, 10 mM forskolin (Sigma Aldrich), 20 ng/ml BDNF (PeproTech), 20 ng/ml GDNF (PeproTech), and 200 nM ascorbic acid (Sigma-Aldrich). The differentiation medium was supplemented with 5 μM Gö6983 (days 1–5), followed by 1 mM dorsomorphin (Sigma Aldrich; days 6–10) and 10 ng/ml NT3 (PeproTech; days 6–19). Culture medium was completely replenished every other day. At Day 14 or Day 19 of differentiation, cells were fixed and processed for immunocytochemistry.

### 2.3. qPCR

Total RNA was isolated from HDFs and hiDFPs from each line at the end of reprogramming using the NucleoSpin RNA kit (Macherey Nagel). cDNA was synthesized from total RNA using Superscript IV reverse transcriptase (Life Technologies). Duplex qPCR reactions were performed in triplicate for each sample using the TaqMan^®^sysytem (Applied Biosystems) with ribosomal 18 S rRNA as the internal standard. An equivalent of 4 ng mRNA per reaction was used. Fold change in gene expression is presented as ΔΔCT relative to HDFs. TaqMan assays used were as follows:

• RANBP17 Hs00910567_m1
• LAMA3 Hs00165042_m1
• PCDH10 Hs00252974_s1
• CDKN2A Hs00923894_m1
• CDKN1A Hs00355782_m1
• TERT Hs00972650_m1
• TERC Hs03454202_s1

### 2.4. Relative telomere length

Quantitative RT-PCR was used to determine changes in the telomere length of hiDFPs and hiPSCs relative to their corresponding HDFs. Total DNA was isolated from HDFs and hiDFPs from each line at the end of reprogramming using the NucleoSpin Tissue DNA kit (Macherey Nagel).

Relative Human Telomere Length Quantification qPCR assay (ScienCell) was used to determine relative telomere length. Two separate PCR reactions were performed in triplicate for each sample using telomere or SCR primer sets and PowerTrack^TM^ SYBR Green Master Mix. An equivalent of 4 ng genomic DNA per reaction was used, in a total of 20 μl per well. Amplification was performed under the following conditions: denaturation for 10 min at 95°C, followed by 32 cycles of denaturation for 20 s at 95°C, annealing for 20 s at 52°C and extension for 45 s at 72°C. The relative telomere length was calculated according to the instructions in the manufacturer’s protocol.

### 2.5. Senescence assay

CellEvent^TM^ Senescence Green Detection Kit (Invitrogen) was used. Following 3 weeks of reprogramming, the cells were plated on Geltrex (Thermo Fisher Scientific)-coated coverslips and incubated overnight. The following day, the assay was performed according to the manufacturer’s protocol. Briefly, cells were washed with PBS and fixed in 4% paraformaldehyde for 10 min. Next, cells were washed with 1% BSA in PBS and incubated with a working solution containing CellEvent^TM^ Senescence Green Probe for 2 h at 37°C without CO_2_, protected from light. After the incubation the plate was read using the EnSight Multimode Plate Reader (PerkinElmer) with fluorescence excitation and emission maxima 490 nm/514 nm.

### 2.6. MitoSOX assay

MitoSOX^TM^ Red mitochondrial superoxide indicator for live-cell imaging (Invitrogen) was used. Following 3 weeks of reprogramming, the cells were plated on Geltrex-coated coverslips and incubated overnight. The following day, the assay was performed according to the manufacturer’s protocol. Briefly, cells were washed with PBS and incubated with 5 μM MitoSOX^TM^ reagent for 10 min at 37°C protected from light. The plate was read using the EnSight Multimode Plate Reader (PerkinElmer) with fluorescence excitation and emission maxima 510 nm/580 nm.

### 2.7. DNA methylation

The amount of methylated DNA was quantified using the Methylated DNA Quantification Kit (Abcam) which specifically measures the level of 5-methylcytosine (5-mC) from total gDNA isolated from hiPSCs, HDFs, and hiDFPs using the NucleoSpin Tissue DNA kit (Macherey Nagel). The assay was conducted as per the manufacturer’s protocol. Briefly, 100 ng of DNA was loaded for each sample in a 96-well strip plate with standards and a negative control. After binding by incubation at 37°C for 90 min, a capture antibody was added to each well and incubated at room temperature for 60 min, followed by incubation for 30 min in the detection antibody. The signal was enhanced by incubation for 30 min in an enhancer solution, followed by a 10 min incubation in a developer solution. The enzymatic reaction was stopped by the addition of a stop solution and the absorbance in each well was then read on a microplate reader at 450 nm. DNA methylation was determined as measured by the production of 5-mC (ng) from total DNA.

### 2.8. Immunocytochemistry and neuron morphological characterization

Cells fixed in 4% paraformaldehyde at 4°C for 10 min were washed in 1× phosphate-buffered saline (PBS), and then permeabilized in PBS with 0.5% Triton X-100 for 5 min at room temperature (RT). Cells were then blocked with 3% goat serum in PBS containing the primary antibody TUJ1 (anti-rabbit, 1:500; Abcam) and incubated overnight at 4°C. Cells were then washed with PBS and incubated with Alexa Fluor^TM^ goat anti-rabbit 594 sary antibody for 1 h at RT. Individual cell nuclei were confirmed using Prolong Diamond antifade mountant with 4’,6-diamidino-2-phenylindole (DAPI; Life Technologies). Imaging was undertaken on a Nikon TE2000E inverted microscope (Nikon) equipped with a Nikon DS-Ri camera. Images were converted to greyscale in ImageJ, scaled and the cell soma size measured using the polygonal selection tool. Neurite number, neurite length and the number of branch points were determined using the NeuronJ plugin in Fiji. Morphological characterization was undertaken from 30 cells per donor age group.

### 2.9. Statistical analysis

Statistical analyses were performed using IBM SPSS Statistics. A two-way mixed ANOVA was used to determine the effect of reprogramming and/or age on senescence, mitochondrial superoxide activity, and the amount of methylated DNA. A one-way ANOVA was used for comparison of hiPSCs and age on gene expression and amount of methylated DNA. A two-way ANOVA was used to compare hiDFP-derived neuron morphologies across donor age groups at two differentiation time points, Day 14 and Day 19. *Post-hoc* analyses were performed with Tukey’s test after confirming the homogeneity of variances. All data are presented as mean ± SEM. Results were considered significant if *p* < 0.05.

## 3. Results

### 3.1. Reprogramming of human dermal fibroblasts to induced dorsal forebrain precursor cells enhances mitochondrial superoxide expression

**Figure 1:**
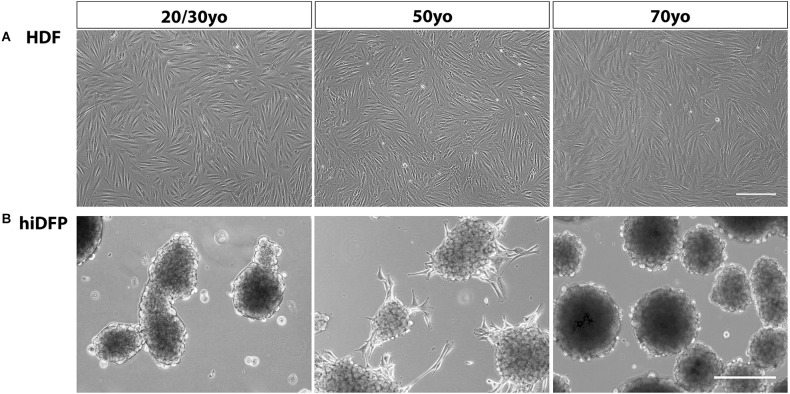
Phase contrast images of **(A)** HDFs and **(B)** resulting hiDFP colonies from 20/30-, 50- and 70-year-old subjects. Scale = 300 μm. HDF, human dermal fibroblast; hiDFP, human induced dorsal forebrain precursor.

Adult human dermal fibroblasts were transfected over four consecutive cycles with *SOX2* and *PAX6* cmRNA and cultured in GYN cortical reprogramming medium. Twenty-one days following the last transfection, we observed distinct morphological changes in the cell lines characterized by the progressive formation of semi-adherent neurosphere-like colonies comprised of neural precursor cells (Figure 1). We did not observe any difference in hiDFP generation with donor age. The conversion efficiency of the cultures was determined by quantifying the number of hiDFPs generated from the number of originally seeded HDFs and ranged from 35% to 68% efficiency, with no effect of age.

**Figure 2:**
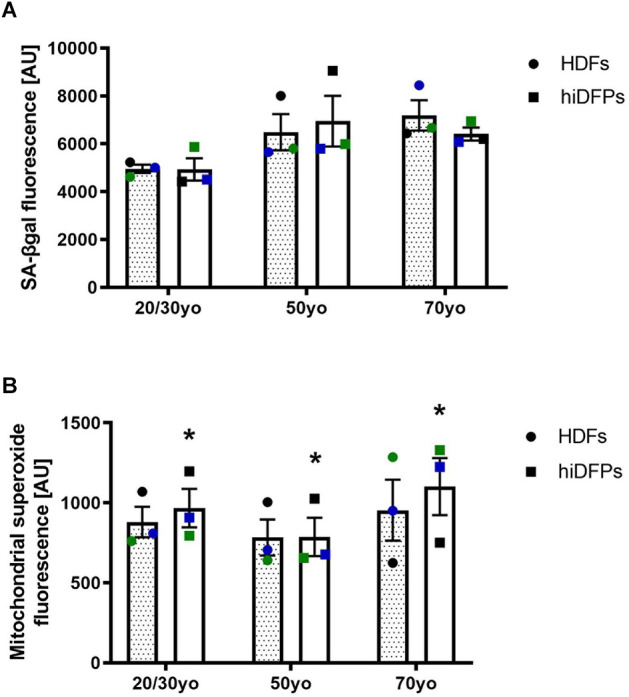
Reprogrammed hiDFPs maintain aging-associated features. **(A)** SA-βgal activity assay of HDFs and hiDFPs. No statistical significance was determined by two-way mixed ANOVA. **(B)** Mitochondrial superoxide levels in HDFs and hiDFPs. Data represent mean ± SEM with *n* = 3. Simple main effect of reprogramming on mitochondrial superoxide levels determined by two-way mixed ANOVA with * for *p* < 0.05.

To address the aspects of cellular aging and its reversal during reprogramming, we used a set of markers including senescence-associated beta-galactosidase (SA-βgal) activity and mitochondrial superoxide. Both of these have previously been shown not to be maintained in hiPSCs (Suhr et al., 2010; Lapasset et al., 2011). When we compared HDFs and corresponding hiDFPs across 3 different age groups, we found no difference in the SA-βgal activity (Figure 2A). However, reprogramming of HDFs to hiDFPs resulted in higher levels of mitochondrial superoxide irrespective of age group (*F*_(1, 6)_ = 10.09, *p* = 0.019, Figure 2B).

### 3.2. Reprogrammed hiDFPs maintain age-associated gene expression

**Figure 3:**
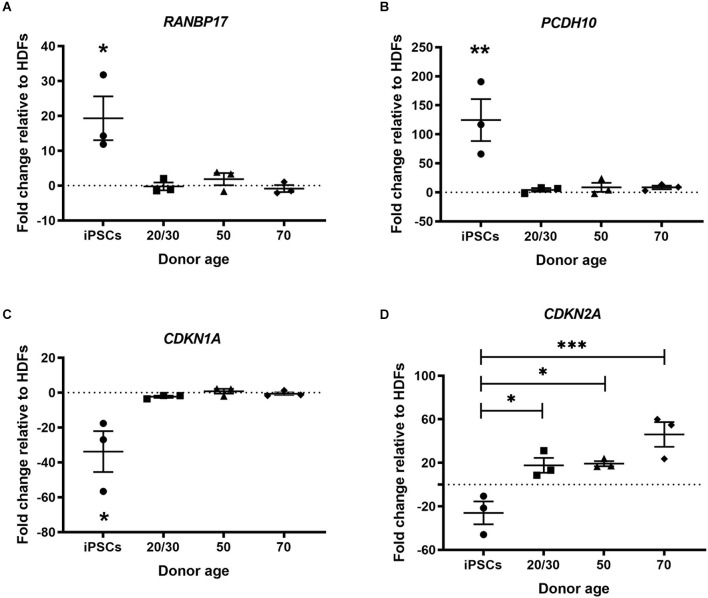
hiPSC reprogramming reverts aging-associated changes in gene expression, while reprogrammed hiDFPs maintain gene expression. **(A)** *RANBP17*; **(B)** *PCDH10*; **(C)** *CDKN1A*; **(D)** *CDKN2A*. Data represent mean ± SEM with *n* = 3. Significance was determined by one-way ANOVA with Tukey *post-hoc* test, with * for *p* < 0.05, ** for *p* ≤ 0.01, and *** for *p* ≤ 0.001.

Multiple genes have been found to change during normal human aging. Many of these genes are restored in hiPSCs revealing a process of rejuvenation. We selected genes based on previous publications to investigate whether cmRNA-mediated direct reprogramming has an effect on aging memory in hiDFPs (De Magalhäes et al., 2009; Mertens et al., 2015). Nuclear transport receptor *RANBP17* is decreased in aged human cells and induced neurons (iNs) with expression restored in hiPSCs (Mertens et al., 2015). Our results confirmed an up-regulation in the expression of *RANBP17* in hiPSCs relative to HDFs, while the expression of *RANBP17* was unchanged in the hiDFPs relative to corresponding HDFs (Figure 3A). Expression of *RANBP17* was significantly increased in hiPSCs compared to hiDFPs (*p* = 0.014, 0.025, and 0.012 for iPSC compared to 20/30yo (years old), 50yo, and 70yo hiDFPs respectively; Figure 3A).

*PCDH10* which encodes the cadherin-related neuronal receptor thought to function in the establishment of specific cell-cell connections in the brain, also decreases with age. We found the expression of *PCDH10* was up-regulated in hiPSCs relative to HDFs but remained unchanged in hiDFPs (Figure 3B). Expression of *PCDH10* was significantly increased in hiPSCs compared to hiDFPs (*p* = 0.008, 0.01, and 0.01 for iPSC compared to 20/30yo, 50yo, and 70yo hiDFPs respectively; Figure 3B).

We investigated two cyclin-dependent kinase inhibitors, *CDKN1A* (p21^Cip1^) and *CDKN2A* (p16^INK4a^). Expression of these genes induces senescence. We observed a downregulation of both *CDKN1A* and *CDKN2A* in hiPSCs relative to HDFs (Figures 3C,D). In contrast, the expression of both *CDKN1A* and *CDKN2A* was maintained in directly reprogrammed hiDFPs (Figures 3C,D). Both *CDKN1A* and *CDKN2A* were significantly reduced in hiPSCs compared to hiDFPs (*CDKN1A: p* = 0.024, 0.014, and 0.018 for iPSC compared to 20/30yo, 50yo and 70yo hiDFPs respectively; *CDKN2A: p* = 0.028, 0.023 and 0.001 for iPSC compared to 20/30yo, 50yo, and 70yo hiDFPs respectively; Figures 3C,D).

### 3.3. Reprogrammed hiDFPs retain telomere length and gene expression

**Figure 4:**
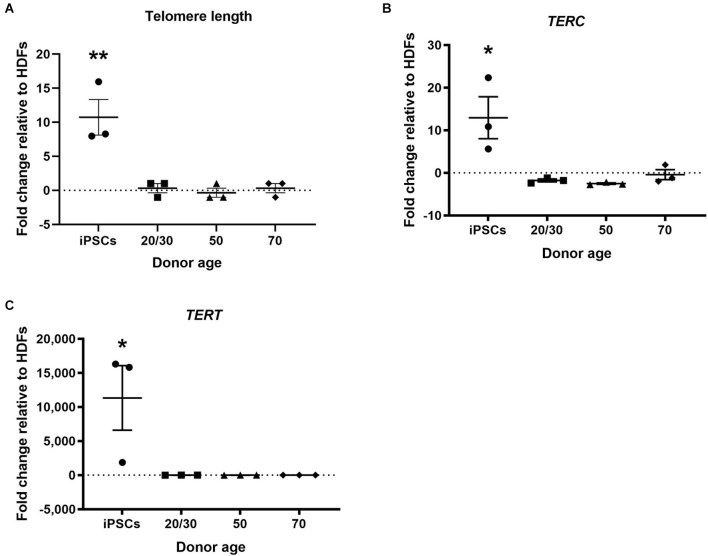
hiPSC reprogramming reverts aging-associated changes in telomere length and gene expression, while reprogrammed hiDFPs maintain expression. **(A)** Relative telomere length; **(B)** *TERC*; **(C)** *TERT*. Data represent mean ± SEM with *n* = 3. Significance was determined by one-way ANOVA with Tukey *post-hoc* test with * for *p* < 0.05, ** for *p* ≤ 0.01.

Telomere shortening is associated with increased age (Harley et al., 1990). Telomerase activity has been found to be upregulated in hiPSCs and the telomeres have been shown to be elongated following the reprogramming process (Marión et al., 2009). In the current study, hiPSCs showed increased relative telomere length to HDFs. However, hiDFPs showed no change in telomere length following reprogramming relative to HDF (Figure 4A). Telomere length was significantly increased in hiPSCs compared to hiDFPs (*p* = 0.004, 0.003, and 0.004 for iPSC compared to 20/30yo, 50yo and 70yo hiDFPs respectively; Figure 4A). We additionally analyzed the expression of the genes associated with telomere length, telomerase RNA component *TERC*, and telomerase reverse transcriptase *TERT*. Both *TERC* and *TERT* have been shown to increase in hiPSCs following reprogramming. This was also observed in the current study (Figures 4B,C). In contrast, cmRNA-mediated direct reprogramming of HDFs to hiDFPs had no effect on the expression of either *TERC* or *TERT* (Figures 4B,C). Both *TERC* and *TERT* expression was significantly increased in hiPSCs compared to hiDFPs (*TERC: p* = 0.015, 0.011, and 0.024 for iPSC compared to 20/30yo, 50yo, and 70yo hiDFPs respectively; *TERT: p* = 0.039, 0.039, and 0.039 for iPSC compared to 20/30yo, 50yo, and 70yo hiDFPs respectively; Figures 4B,C).

### 3.4. Reprogramming of human dermal fibroblasts to induced dorsal forebrain precursor cells enhances DNA methylation

**Figure 5:**
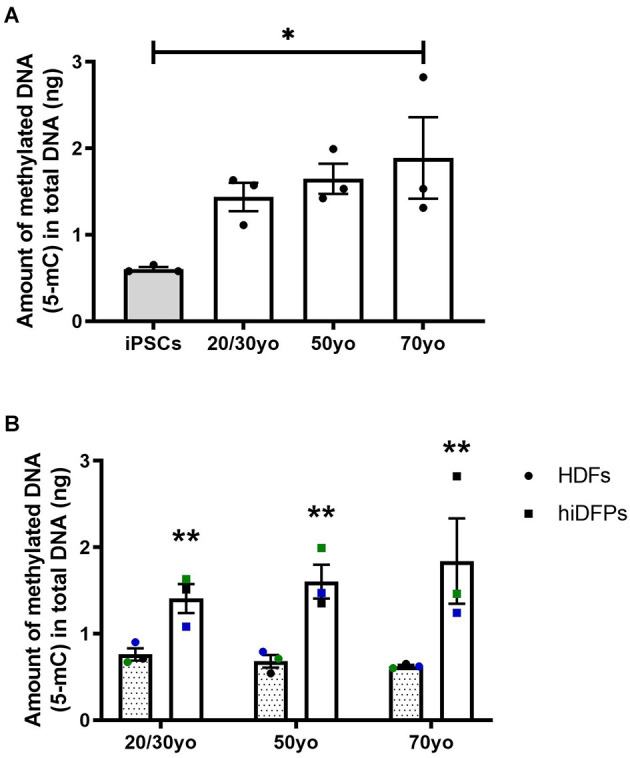
Reprogrammed hiDFPs exhibit increased amounts of DNA methylation as measured by the production of 5-methylcytosine (5-mC). **(A)** Amount of methylated DNA in hiPSCs and hiDFPs relative to HDF. Significance was determined by one-way ANOVA with Tukey *post-hoc* test. **(B)** Amount of methylated DNA in HDFs and hiDFPs. A simple main effect of reprogramming irrespective of age group was determined by two-way mixed ANOVA. Data represent mean ± SEM with *n* = 3. * for *p* < 0.05, ** for *p* ≤ 0.01.

It has been established that DNA methylation biomarkers can determine the biological age of any tissue across the entire human lifespan (Horvath, 2013; Salameh et al., 2020). In regards to cell reprogramming, Horvath (2013) demonstrated that hiPSCs exhibit a DNA methylation age between −1 and 0 years, equivalent to embryonic stem cells. In the current study, we observed the amount of methylated DNA was increased in 70yo hiDFPs compared to hiPSCs (*p* = 0.036; Figure 5A). While the amount of methylated DNA in the 20/30yo and 50yo hiDFPs was not significantly different to hiPSCs, an upward trend was observed. Interestingly, the amount of DNA methylation was significantly increased in hiDFPs compared to their parental HDFs irrespective of age group (*F*_(1, 6)_ = 24.13, *p* = 0.003; Figure 5B).

### 3.5. Morphology of hiDFP-derived neurons from different donor ages

**Figure 6:**
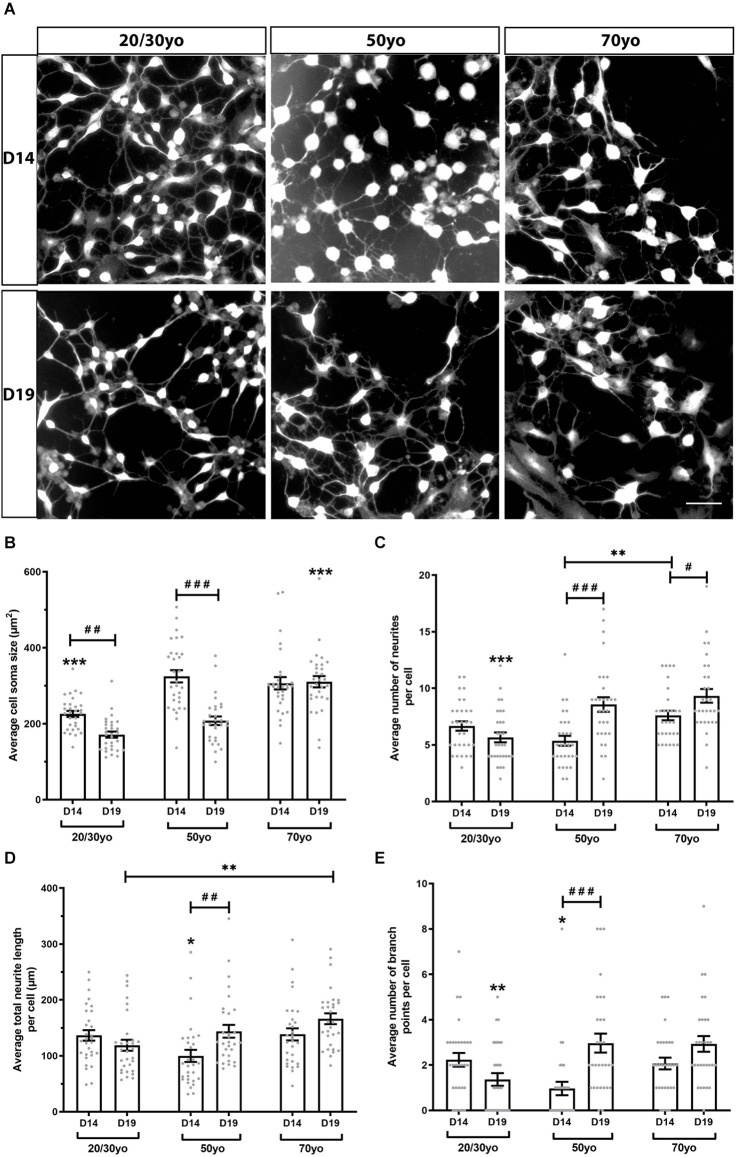
hiDFP-derived neurons exhibit complex neuronal morphologies displaying enhanced complexity with age. **(A)** Representative images of TUJ1+ hiDFP-derived neurons for each donor age group and at two differentiation time points Day 14 (D14) and Day 19 (D19). Scale: 50 μm. **(B)** Average cell soma size. **(C)** Average number of neurites per cell. **(D)** Average total neurite length per cell. **(E)** Average number of branch points per cell. **(B–E)** Data represent mean ± SEM with *n* = 30 cells per age group at each time point. Significance was determined by two-way ANOVA with pairwise comparisons following a significant interaction. A significant effect of age group within a given time point (D14 or D19) is denoted by * for *p* < 0.05, ** for *p* < 0.01, *** for *p* ≤ 0.001. A significant effect of differentiation time point (comparison of D14 to D19) within each age group is denoted by ^**#**^ for *p* < 0.05, ^**##**^ for *p* < 0.01, ^**###**^ for *p* ≤ 0.001.

To investigate whether increased donor age resulted in altered neuronal morphology when hiDFPs were differentiated into neurons, we undertook Scholl analysis of hiDFP-derived TUJ1-positive neurons at two differentiation time points, Day 14 (D14) and Day 19 (D19; Figure 6). We observed that hiDFPs-derived neurons exhibited complex neuronal morphologies (Figure 6A). Neurons that were differentiated for 14 days (D14) had a significantly smaller average cell soma size when derived from 20/30yo donors than 50yo or 70yo donors, while Day 19 (D19) neurons had a significantly larger average cell soma size when derived from 70yo donors (*p* = 0.000048 and *p* = 0.00000019, respectively; Figure 6B). Prolonged differentiation from D14 to D19 resulted in a reduction in average cell soma size in 20/30yo neurons (*p* = 0.003) and 50yo neurons (*p* = 0.0000000011), but no difference in average cell soma size in 70yo neurons (*p* = 0.824; Figure 6B). However, the average number of neurites per cell increased with prolonged differentiation to D19 in 50yo neurons (*p* = 0.000012) and 70yo neurons (*p* = 0.016), but not 20/30yo neurons (*p* = 0.161; Figure 6C). Similarly, within neurons differentiated to D19, the 20/30yo age group displayed the smallest average number of neurites per cell when compared to 50yo and 70yo (*p* = 0.000202; Figure 6C). The average total neurite length per cell did not change with prolonged differentiation within each age group, except for the 50yo cells (*p* = 0.003), but in neurons differentiated for 19 days, the 70yo neurons showed a greater average total neurite length per cell than the 20/30yo counterparts (*p* = 0.004; Figure 6D). The average number of branch points per cell was no different within each age group with prolonged differentiation to D19, except in the 50yo age group (*p* = 0.000018; Figure 6E). Amongst neurons that were differentiated to D19, those derived from the 20/30yo donors showed a smaller average number of branch points per cell (*p* = 0.002; Figure 6E). These results indicate that neuronal morphology is altered in hiDFP-derived neurons with increasing donor age.

## 4. Discussion

The current study has demonstrated for the first time that chemically modified mRNA (cmRNA)-mediated reprogramming of adult human dermal fibroblasts (HDFs) to human induced dorsal forebrain precursors (hiDFPs) prevents cell rejuvenation and maintains the aging memory of the donor HDFs. The use of our cmRNA hiDFP reprogramming technology, therefore, provides an enhanced mechanism by which to model neurological diseases of aging than the current use of hiPSCs.

Multiple studies have previously shown that reprogramming of HDFs to hiPSCs resets aspects of cellular and molecular aging (Marión et al., 2009; Suhr et al., 2009, 2010; Horvath, 2013; Miller et al., 2013). This rejuvenation has a significant effect on the potential use of hiPSCs to model diseases associated with aging. Since aging is required for the development of late-onset diseases, multiple approaches have been proposed which can induce “aging” in the hiPSC-derived cultures to allow for the resurgence of disease pathology. The aging process involves a number of cellular changes that can contribute to the development of late-onset diseases. These include accumulation of misfolded proteins, mitochondrial dysfunction, or increased levels of ROS. Additionally, external factors such as lifestyle or exposure to chemicals (Chin-Chan et al., 2015) are linked to disease risk and trigger age-related changes. Multiple studies have drawn from these triggers and attempted to mimic stress-induced changes associated with normal aging to model late-onset diseases in hiPSCs. These studies included exposure to hydrogen peroxide (ROS production) or CCCP and rotenone (mitochondrial stress; Neely et al., 2017; Zhang et al., 2020; Liedtke et al., 2022).

The specific pathways that can be targeted can also include strategies to shorten telomeres, induce heterochromatin loss, mitochondrial dysfunction, or induction of senescence and accumulation of misfolded proteins (López-Otín et al., 2013). Another approach that has been investigated involved genetically triggering aging features in hiPSC-derived cells using premature aging syndrome HGPS (Miller et al., 2013). This method involved over-expression of progerin in hiPSC-derived dopamine neurons to model Parkinson’s disease. While these proposed cellular aging strategies can be used in order to obtain “age”-appropriate cell types from hiPSCs, these approaches rely on the concept that aging can be “induced” by one key trigger. This may be correct for modeling of genetic conditions such as progeria, but normal aging does not follow such a simple route.

Alternatively, direct reprogramming of HDFs to a mature neuron fate (iN), bypassing the initial generation of hiPSCs, retains the aging memory of cells (Mertens et al., 2015; Yang et al., 2015; Tang et al., 2017). This confirms that over-expression of the Yamanaka factors results in the removal of aging signatures in hiPSCs. However, while iNs provide a strategy by which to generate authentic human neurons that reflect important aspects of cellular age and provide a viable tool for neurological disease modeling, the generated neurons are post-mitotic and cannot be expanded for high throughput analysis. In contrast, our cmRNA-mediated reprogramming strategy generates hiDFPs with the capability of generating cortical neurons. We have demonstrated that our technology has no effect on the expression of aging markers and telomere length. In particular, we observed the senescent-associated gene *CDKN2A* was increased 20–40 fold in hiDFPs across all age groups with respect to hiPSCs. *CDKN2A* is one of the most intensively investigated markers of aging and senescence. *CDKN2* expression is absent during embryonic development but is highly expressed in advanced age and senescence (Suhr et al., 2010; Wagner and Wagner, 2022). Up-regulation of *CDKN2A* in directly reprogrammed hiDFPs demonstrates maintenance of age not observed in hiPSCs and indicates that direct reprogramming retains the chromatin age state.

On investigating the effect of donor age on neuronal morphology, we observed that following 19 days of differentiation hiDFP-derived neurons generated from 70yo donors exhibited a more complex morphology than neurons generated from 20/30yo donors. Neurons from 70yo donors displayed a larger cell soma size, a greater number of neurites per cell as well as longer neurite length and a greater number of branch points than neurons generated from 20/30yo donors. Very little is known regarding the effect of age on the morphology of human neurons. While a wide loss of neurons is not present in normal aging, the principal age-related alteration in neuronal morphology involves a reduction in neurite length and number (Castelli et al., 2019). This however was not seen in our directly reprogrammed hiDFP-derived neurons. Samsonovich and Ascoli (2006) demonstrated that the size of neurites may be under internal homeostatic control. In particular, fluctuations in neurite number, length, or membrane area in one part of a neuron tend to be counterbalanced in other parts of the same neurons. Furthermore, previous findings in *C. elegans* have demonstrated that healthy neurons exhibit novel neurite outgrowth with increasing age (Toth et al., 2012; Hess et al., 2019), possibly the result of synaptic deterioration and dendritic reorganization. We propose more detailed investigation is required to determine the mechanism by which altered morphology occurs in human neurons with aging.

Interestingly, we did observe an increase in the level of mitochondrial ROS production in hiDFPs. During aging, mitochondria begin to produce less ATP and more ROS, thought to result in an energetic crisis that triggers the initiation of neurodegenerative diseases and accelerates aging (Cen et al., 2021; Ebanks and Chakrabarti, 2022). However, it has also been shown that ROS plays a role in regulating activity-dependent synaptic structural plasticity (Oswald et al., 2018) and neuroprotection (Huang and McNamara, 2012). In addition, we observed an increase in the amount of DNA methylation in hiDFPs compared to their parental HDFs. This is in contrast to the effect of epigenetic rejuvenation seen in hiPSCs (Horvath, 2013) and indicates that our direct-to-hiDFP reprogramming protocol has an effect of promoting DNA methylation status. While our current findings cannot delineate the cause of the increased ROS production or DNA methylation in hiDFPs, it is apparent this is a response to the mechanism of reprogramming and is independent of age.

Overall, our findings confirm that cmRNA-reprogrammed hiDFPs provide an alternative strategy for modeling late-onset neurodegenerative diseases and show age-associated phenotypes not normally seen in hiPSC-derived cultures. With the increase in aging populations around the world, the development of reliable models for age-related neurodegenerative diseases, such as Alzheimer’s or Parkinson’s disease, is becoming crucial. A human cell culture system retaining aging features as demonstrated by our cmRNA-reprogrammed hiDFPs will significantly facilitate our understanding of neurodegenerative disease and allow for more efficient identification of biomarkers and new therapeutic targets.

## Data availability statement

The raw data supporting the conclusions of this article will be made available by the authors, without undue reservation.

## Author contributions

AM-C: resources, methodology, validation, formal analysis, investigation, writing—final version, review and editing. MT-W: funding acquisition, methodology, validation, formal analysis, investigation, writing—original draft. CC: resources, methodology, investigation, and formal analysis. NE: resources and methodology. KJ: conceptualization, methodology, and investigation. BC: conceptualization, validation, writing—final version, review and editing, and supervision. All authors contributed to the article and approved the submitted version.
